# Rare Case of Tracheal Fibromyxoma in Obstructive Tracheal Mass

**DOI:** 10.7759/cureus.24471

**Published:** 2022-04-25

**Authors:** David R Mann, Kunal J Patel, Tiffany Baker, Barry C Gibney

**Affiliations:** 1 General Surgery, Medical University of South Carolina, Charleston, USA; 2 Cardiothoracic Surgery, University of Virginia, Charlottesville, USA; 3 Pathology, Medical University of South Carolina, Charleston, USA; 4 Thoracic Surgery, Medical University of South Carolina, Charleston, USA

**Keywords:** partial airway obstruction, tracheal resection, neoplasms of trachea, rare benign tumor, tracheal tumor

## Abstract

A 77-year-old male with a history of chronic obstructive pulmonary disease (COPD) who presented with cough, congestion, and stridor and was found to have a near obstructing tracheal mass. He subsequently underwent excision of the mass. On pathologic examination, it was diagnosed as fibromyxoma of the trachea. Primary tumors of the trachea are rare, and fibromyxoma of the trachea is extremely rare. This is the third report of a fibromyxoma on the tracheal wall. In this report the clinical manifestations, and surgical management were compared with the other two reported cases.

## Introduction

Primary tumors of the trachea are rare, occurring at an estimated rate of 2.6 new cases per 1,000,000 people per year [1]. In the National Cancer Institute (NCI) Surveillance, Epidemiology, and End Results (SEER) 1972-2004 database, only 578 cases were reported, with 80% being malignant and squamous cell carcinoma being the most predominant type [2]. Benign tracheal tumors have a male predominance and are usually seen in the 5^th^ and 6^th^ decade of life [3]. Given their rarity and subsequent low index of suspicion, there is often difficulty in making the initial diagnosis which can delay intervention. Fibromyxomas are a type of rare benign soft tissue tumor that are derived from mesenchyme and have been previously described on the skin, stomach, digits, and mandible [4-7]. To our knowledge there have only been two other reported primary tracheal fibromyxomas [8,9].

## Case presentation

History

A 77-year-old male with a history of chronic obstructive pulmonary disease (COPD) who presented with chief complaints of worsening cough, dyspnea, congestion, and stridor. His symptoms had persisted despite using his prescribed COPD inhalers. He reported smoking for many years but had quit several years prior to presentation. Additional co-morbidities included type 2 diabetes mellitus, hypertension, dyslipidemia, and gastroesophageal reflux disease (GERD).

Examination and hospital course

**Figure 1:**
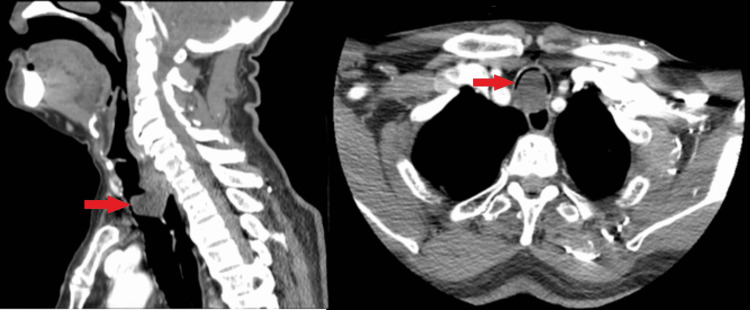
Sagittal and axial computed tomography images of near obstructing tracheal mass

The patient was initially evaluated in the emergency department near his home and treated with steroids, under the presumption of an COPD exacerbation, with no improvement. Ultimately, a computerized tomography (CT) scan was obtained that demonstrated a near obstructing tracheal mass (Figure 1) with mass effect on the cervical esophagus and thickening of the anterior esophageal wall. He was transferred to our facility for further care. On arrival he continued to have stridor and was unable to lie flat secondary to anxiousness. It was unclear to us if his airway was actually compromised when supine or if the sensation of potential breathing difficulties inhibited his ability to tolerate the position. Initial working diagnosis was adenoid cystic carcinoma. Positron emission tomography (PET) was attempted but was aborted when the patient became hypoxic when placed completely supine. Given the size and highly symptomatic nature of the mass, he was offered resection of the tracheal lesion.

Operation

In discussions with our anesthesia colleagues, several concerns were raised. Primarily, the patient was unable to tolerate supine positioning, and he would/did not tolerate performing pre-operative bronchoscopy to evaluate the lesion and airway access for intubation. Out of concern for losing the airway on induction, and questions regarding the efficacy of an emergent surgical airway in the event of airway collapse, we elected to begin the operation by placing the patient on venovenous extracorporeal membrane oxygenation (ECMO) before anesthesia induction. We were then safely able to perform fiberoptic bronchoscopy and secure a 6.0 endotracheal (ET) tube past the mass. We elected to remain on ECMO; however, as a precaution.

Pre-operatively, we had estimated the mass to be 3cm in diameter and that we would need to resect at least 4cm of trachea for a complete excision. As such, we felt it necessary to increase tracheal mobilization, and so a right hilar release was performed.

We then supinated the patient, and a collar incision was made to expose the trachea; however, we ultimately required extension to a median sternotomy due to the distal position of the mass. Utilizing cross-table ventilation at this juncture, the trachea was then circumferentially dissected, and the tumor was resected en bloc with negative gross and frozen margins. The distal trachea was then mobilized to allow for a tension free anastomosis, which was performed over an ET tube. Given the length of the procedure and the patient’s tenuous baseline pulmonary function, we elected to retain the ET tube at the completion of the case; however, we did decannulate the ECMO.

Post-operative course

The patient recovered in the Cardiovascular ICU. He was extubated on postoperative day 1, and his hospital course was significant for acute blood loss anemia requiring blood transfusion, dysphagia, and aspiration requiring percutaneous endoscopic gastrostomy (PEG) tube placement for enteral nutrition. He was discharged on postoperative day 15. He was noted to be clinically improved when seen in the clinic for his one-month post-operative visit. The patient reported resolution of his dyspnea, stridor, and dysphagia and was maintaining weight on his oral intake alone. He was seen again at four months, and his PEG tube was removed. Since his pathology was benign, no surveillance imaging was necessary, and a clinic visit was scheduled for six months to follow his progress. The patient was subsequently lost to follow-up.

Histopathologic findings

**Figure 2:**
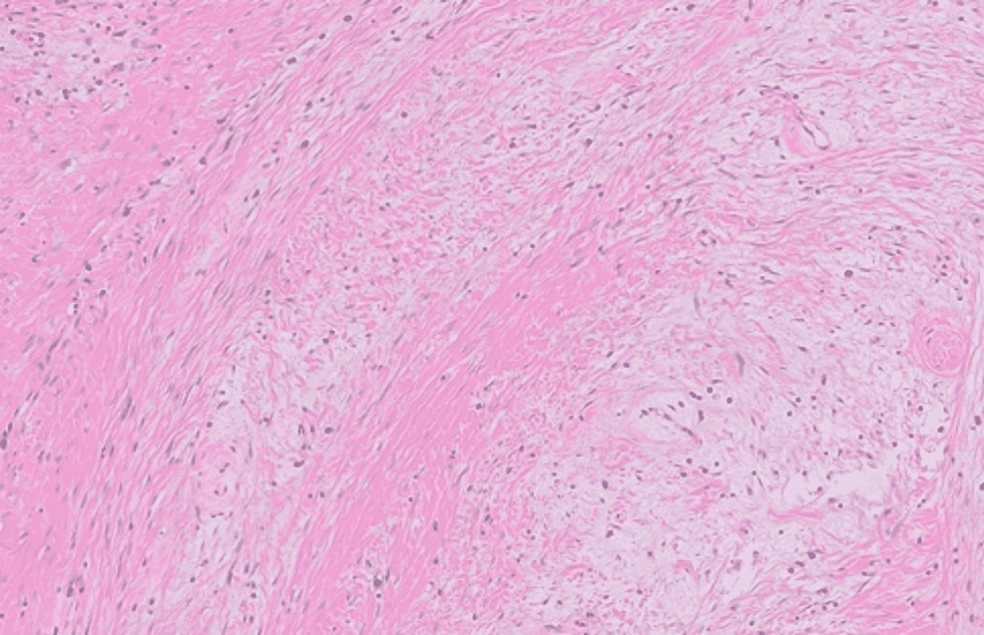
Photomicrograph of H&E stained tissue section at 100X original magnification Hematoxylin and eosin (H&E) stained tissue sections demonstrated a bland fibrous tumor with alternating zones of high- and low-density collagen. Some of the low-density areas contain myxoid material. The lesion is predominantly comprised of bland spindle cells. No mitoses or necrosis are identified. The accompanying, scattered inflammatory infiltrate comprised of plasma cells and lymphocytes. A ki-67 immunohistochemical stain reacts with less than 1% of tumor nuclei, indicating a low proliferation rate. The differential diagnosis for this benign fibromyxoid lesion includes, fibroma and fibromyxoma. Pertinent negative immunohistochemical stains include SOX-10, EMA, ALK-1, MUC-4, beta-catenin, and IgG4. Their observed patterns or complete lack of reactivity render unlikely or rule out the following diagnoses: peripheral nerve sheath tumor, inflammatory myofibroblastic tumor, low grade fibromyxoid sarcoma, fibromatosis, and IgG4 sclerosing disease.

The tumor measuring 3.1 x 2.1 x 1.7 cm was sectioned and examined. It was noted on examination to occlude greater than 90% of the tracheal lumen. Upon further examination the tumor revealed a benign appearing histomorphology and a low proliferation index based on Ki-67 immunohistochemistry comprised predominantly of bland spindled cells and collagen with myxoid change and a lymphoplasmacytic infiltrate (Figure 2). No malignant features were evident such as pleomorphism or mitoses. The final diagnosis was made of fibromyxoma based on myxoid changes seen on pathologic examination.

## Discussion

Benign tracheal tumors are rare and include leiomyoma, fibroma, fibrohistiocytoma, fibromyxoma, and benign peripheral nerve sheath tumors. A review of the literature revealed only two other cases of fibromyxoma have been reported in the literature [8, 9]. These tumors have been described as round and with a smooth surface appearance and are often pedunculated. These lesions are small and are most commonly asymptomatic. However, with increasing size these may cause stridor or be misdiagnosed as bronchial asthma [9]. Half of those benign tumors are located in the lower third of the trachea [10]. The etiology of these tumors is poorly understood.

The other two reported cases of tracheal fibromyxoma were treated with endoscopic removal with biopsy forceps [8] and a surgical mid-tracheal sleeve resection [9], respectively. The endoscopically removed tumor measured 1 mm in size, while the surgically resected was reportedly large enough to restrict airflow and cause dyspnea and wheezing [8,9]. Other techniques include microwave (MW), argon plasma coagulation (APC), high radiofrequency snares, and high-powered laser irradiation with neodymium-yttrium-aluminum-garnet (Nd-YAG) laser, and dehydrated ethanol injection therapy through rigid and flexible bronchoscopy [11]. 

Careful consideration should be given when planning an intervention for benign tracheal tumors. Endoscopic tumor removal can often be appropriate when tumor characteristics are favorable (strictly endoluminal, limited extent within the endobronchial tree, low probability of recurrence) or when surgery is contraindicated due to inadequate pulmonary function or poor general status [12]. Indications for surgical resection include imminent airway obstruction, concurrent organizing pneumonia, significant bronchodilation beyond the tumor, when malignancy cannot be ruled-out histologically, extensive tracheobronchial wall involvement, invasion of the tracheal wall or surrounding structures, and when fatal hemorrhage might be caused by perforation [12]. In this patient, the presence of a highly symptomatic tumor with impending airway obstruction and CT imaging concerning for significant tumor involvement of the tracheal wall was the primary consideration when planning an intervention. In addition, there was radiographic evidence mass effect on the cervical esophagus with thickening of the esophageal wall, which could be consistent with malignancy. 

## Conclusions

Benign tracheal tumors continue to prove a difficult diagnostic challenge given their insidious disease progression. Although low on the differential when evaluating patients with airway obstruction, they can prove to be a dangerous pathology with near-complete tracheal obstruction, as was the case in our patient. Particularly, large lesions make operative planning more complex, as we encountered here. They also may impinge on several critical paratracheal structures that can make resection and reconstruction difficult. The decision as to whether to proceed with endobronchial removal or surgical resection continues to be dependent on patient and tumor characteristics. Small, completely endoluminal tumors, with limited extent in the tracheobronchial tree and low risk of recurrence and poor surgical candidates may benefit most from endobronchial removal and avoid the morbidity of surgical removal and reconstruction. As more cases are reported, the etiology and optimal management of these tumors will be elucidated.
